# Effects of oral cystine and glutamine on exercise-induced changes in gastrointestinal permeability and damage markers in young men

**DOI:** 10.1007/s00394-022-02806-1

**Published:** 2022-02-01

**Authors:** Yusei Tataka, Miki Haramura, Yuka Hamada, Miho Ono, Sakiko Toyoda, Toshiyuki Yamada, Ayano Hiratsu, Katsuhiko Suzuki, Masashi Miyashita

**Affiliations:** 1 Graduate School of Sport Sciences, Waseda University, Saitama, Japan; 2 Waseda Institute for Sport Sciences, Waseda University, Saitama, Japan; 3 Institute of Food Sciences and Technologies, Ajinomoto Co., Inc., Kanagawa, Japan; 4 Research Institute for Bioscience Products & Fine Chemicals, Ajinomoto Co., Inc., Kanagawa, Japan; 5 Sports Nutrition Department, Ajinomoto Co., Inc., Tokyo, Japan; 6 Faculty of Sport Sciences, Waseda University, 2-579-15 Mikajima, Tokorozawa, Saitama 359-1192 Japan

**Keywords:** Exercise-induced gastrointestinal syndrome, Amino acids supplementation, Strenuous running, Lactulose:mannitol, Intestinal fatty acid-binding protein

## Abstract

**Purpose:**

Although acute prolonged strenuous exercise has been shown to increase markers of gastrointestinal permeability and damage, little is known regarding the efficacy of nutritional supplement interventions on the attenuation of exercise-induced gastrointestinal syndrome. This study addressed the effects of oral amino acid supplementation on markers of gastrointestinal permeability and damage in response to exercise.

**Methods:**

Sixteen active men aged 22.7 ± 2.6 years (mean ± standard deviation) completed placebo or cystine and glutamine supplementation trials in random order. Participants received either a placebo or cystine and glutamine supplements, three times a day for 5 days, separated by a 2-week washout period. On day 6, participants took their designated supplements 30 min before running at a speed corresponding to 75% of maximal oxygen uptake for 1 h, followed by a 4-h rest period. Blood samples were collected pre-exercise, immediately post-exercise, 30 min post-exercise, and 1, 2 and 4 h post-exercise on day 6. The plasma lactulose to mannitol ratio (L:M) and plasma intestinal fatty acid-binding protein (I-FABP) were used as markers of gastrointestinal permeability and damage, respectively.

**Results:**

Plasma L:M (linear mixed model, coefficient ± standard error: − 0.011 ± 0.004, *P* = 0.0090) and changes (i.e., from pre-exercise) in plasma I-FABP (linear mixed model, − 195.3 ± 65.7 coefficient ± standard error (pg/mL), *P* = 0.0035) were lower in the cystine and glutamine supplementation trial than in the placebo trial.

**Conclusion:**

Oral cystine and glutamine supplementation attenuated the markers of gastrointestinal permeability and damage after 1 h of strenuous running in young men.

**Trial registration number:**

UMIN000026008.

**Date of registration:**

13 December 2018.

## Introduction

An acute bout of strenuous exercise has been shown to increase gastrointestinal permeability and intestinal cellular damage, attenuating barrier function and this leads to an initial pro-inflammatory cascade, eventually causing gastrointestinal distress [1–3]. These exercise-induced increases in gastrointestinal permeability and intestinal cellular damage are thought to be mediated via the following two main mechanisms: (1) an increase in core temperature during or shortly after strenuous exercise and/or (2) reduced total splanchnic perfusion, causing gastrointestinal ischemia [4]. Although these changes in gastrointestinal permeability and intestinal cellular damage are transient, it is important to address the chronic consequences of repeated bouts of training sessions that lead to chronic health complications, including chronic inflammatory conditions and fatigue [5].

Some nutritional supplement intervention studies, including bovine colostrum, polyphenols, probiotics, and amino acids have examined the effects of supplementation on exercise-induced increases in gastrointestinal permeability and intestinal cellular damage (for a review of these, see Reference [5]). For instance, glutamine (an amino acid) supplementation has been shown to reduce increased gastrointestinal permeability and intestinal cellular damage from strenuous exercise [6–9]. In addition, cystine (a sulphur-containing amino acid) is the main precursor of glutathione [10], which protects the cells against oxidative stress and is known to inhibit inflammation [11, 12]. Indeed, a recent in vitro study demonstrated that cystine reduced oxidative stress-induced intestinal barrier dysfunction [13]. Collectively, these findings indicate that glutamine and cystine may play important roles in protecting intestinal barrier function via heat and oxidative stress-induced signaling pathways, respectively. However, it remains unknown whether combined glutamine and cystine supplementation can effectively attenuate the exercise-induced increase in gastrointestinal permeability and damage in humans.

Therefore, the purpose of the present study was to examine the effects of oral cystine and glutamine supplementation on markers of gastrointestinal permeability and damage in response to an acute bout of prolonged strenuous exercise in active men. We chose the plasma lactulose to mannitol (L:M) ratio and plasma intestinal fatty acid-binding protein (I-FABP) as markers of gastrointestinal permeability and mucosal damage, respectively. Plasma L:M ratio, not urine L:M ratio, was chosen since the collection of incomplete or inaccurately timed urine samples may lead to errors in estimation of prescribed sugar permeability. Plasma I-FABP was chosen since this is widely used in the previous studies and it reflects a breakdown in endothelial cell integrity (for a review, see Reference [3]). We tested the hypothesis that compared to ingestion of maltodextrin, oral cystine and glutamine supplementation would attenuate exercise-induced increases in plasma L:M and I-FABP concentrations.

## Materials and methods

### Participants

After approval from the Institutional Ethical Advisory Committee (Approval number: 2018–270), 16 young, Japanese men gave written informed consent to participate in this study. This study was registered in advance using the University Hospital Medical Information Network Center, a system for registering clinical trials (ID: UMIN000026008). Exclusion criteria were as fllows: being a current smoker; taking any medication or supplement known to affect lipid and carbohydrate metabolism; major illness, including gastrointestinal disorders or physical problems (acute or chronic), limiting the ability to perform physical activity. Habitual information regarding physical activity were obtained via interviews, while additional questionnaires indicated that all participants met the current international public health guidelines for physical activity (i.e., moderate-intensity physical activity for at least 150 min per week, vigorous-intensity physical activity for 75 min per week or an equivalent combination of moderate and vigorous-intensity physical activity) [14]. The physical characteristics of the participants (mean ± standard deviation (SD)) were as follows: age, 22.7 ± 2.6 years; height, 1.71 ± 0.05 m; body mass, 67.0 ± 8.0 kg; body mass index, 22.8 ± 2.5 kg/m^2^; and maximum oxygen uptake (measured while running on a treadmill) 54.3 ± 8.8 mL/kg/min.

### Anthropometry

Body mass was measured to the nearest 0.1 kg using a digital scale (Inner Scan 50, Tanita Corporation, Tokyo, Japan). Height was measured to the nearest 0.1 cm using a stadiometer (YS-OA, AS One Corporation, Osaka, Japan). Body mass index was calculated as weight in kilogrammes divided by the square of height in metres.

### Preliminary tests

Approximately 14 days before the first main trial, participants underwent two preliminary exercise tests performed on a motorized treadmill (Jog Now 700, Technogym, Cesena, Italy). A 16-min, four-stage, submaximal treadmill test was conducted to determine the relationship between running speed and oxygen uptake. Initial running speed was set between 6.0 and 8.0 km/h depending on each participant’s physical activity level (as determined by the initial screening interview and questionnaire). The treadmill was level throughout the test period, and speed was increased by 1.0 or 1.5 km/h every 4 min, depending on each participant’s physical activity level. Next, maximum oxygen uptake was directly measured using an incremental uphill protocol at a constant speed, until participants reached volitional fatigue [15]. The initial inclination of the treadmill was set at 3.5% for the test. Thereafter, the gradient was increased by 2.5% every 3 min. Heart rate was monitored throughout these tests using short-range telemetry (Polar RCX3, Polar Electro, Kempele, Finland). Oxygen uptake, carbon dioxide production and respiratory exchange ratio were measured using a stationary gas analyzer (Quark RMR, COSMED, Rome, Italy). Ratings of perceived exertion were periodically assessed during tests using the Borg scale [16]. Data generated from these two tests were used to determine the running workload (i.e., running speed) corresponding to 75% of each participant’s maximum oxygen uptake, and this workload was used for the main trials.

Seven days after completing the preliminary exercise tests, one baseline gastrointestinal permeability evaluation was performed (further details are provided under “Evaluation of gastrointestinal permeability”). This baseline evaluation was conducted in a resting state (i.e., without performing exercise) throughout the 6-h evaluation period.

### Study design and protocol

**Fig. 1:**
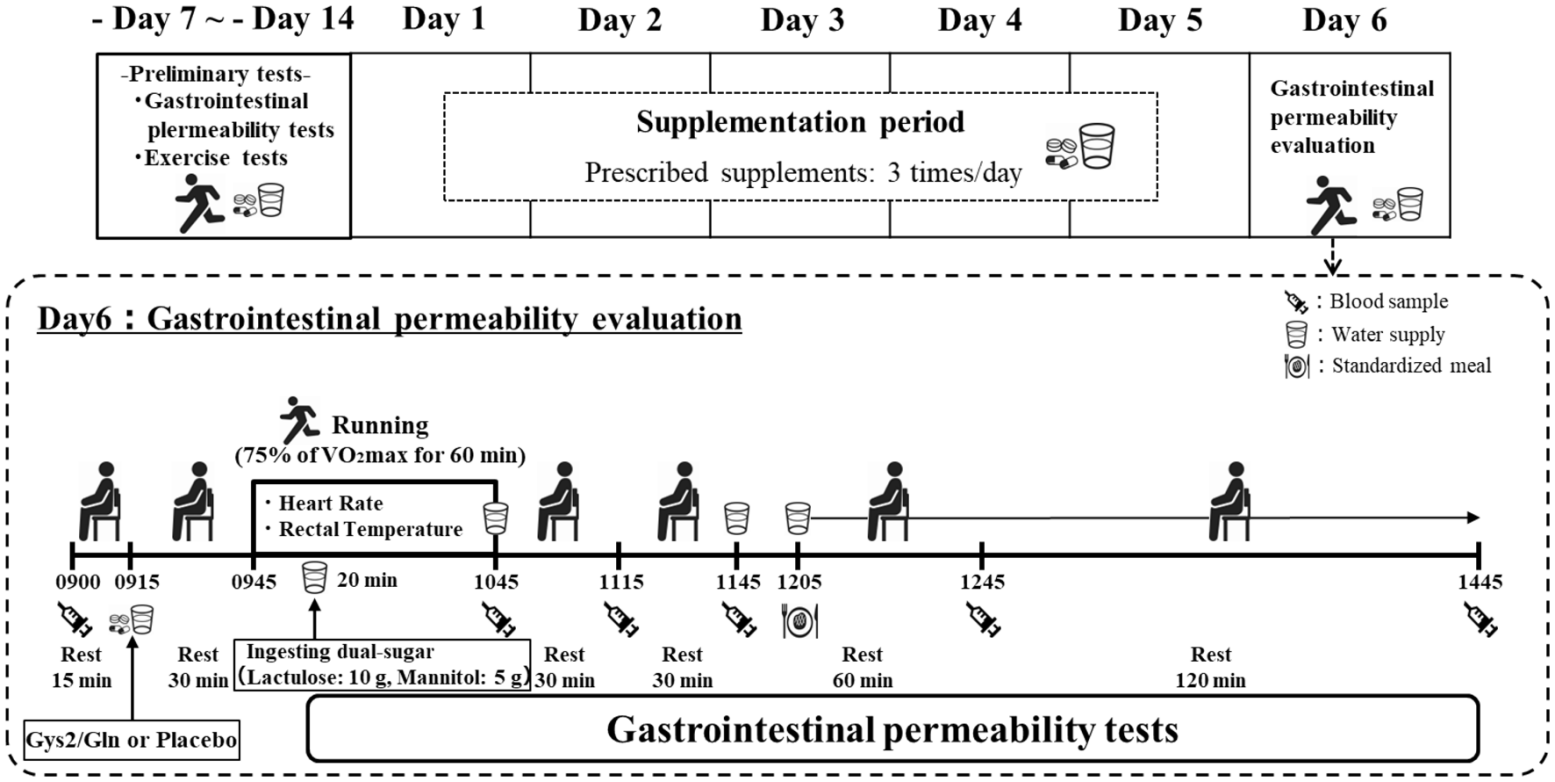
A schematic representation of the study schedule and protocol. *VO2max* maximum oxygen uptake, *Cys2/Gln* cystine and glutamine supplementation

A randomized, double-blind, placebo-controlled crossover design was used in the present study. The primary outcomes of the present study were plasma L:M and I-FABP concentrations. Each participant underwent two, 6-day trials in a randomized, counterbalanced order: placebo or cystine and glutamine supplementation. The interval between trials was 14–22 days. A schematic illustration of the study protocol is presented in Fig. 1. Participants ingested 0.23 g of l-cystine, 1.00 g of L-glutamine and 1.23 g of maltodextrin three times (i.e., at 1000, 1500 and before sleep) per day for 5 days or 2.46 g of maltodextrin three times per day (i.e., at 1000, 1500 and before sleep) for 5 days. This glutamine dose and regimen were chosen since previous studies observed a suppressive effect of glutamine on oxidative stress [17] and no adverse effects in healthy adults when the individuals consumed a higher dose of glutamine than the present study [6]. Also, this cystine dose and regimen were chosen because of its high safety profile and no adverse effects in healthy adults (i.e., 2100 mg/day for 4 weeks) [18]. Participants were instructed to maintain their current physical activity level during the 5-day supplementation period and to refrain from taking any medications or supplements on days 3, 4 and 5. Participants were also instructed to refrain from exercising and to consume three standardized meals (i.e., provided by investigators) on day 5. Furthermore, all participants were informed of the foodstuffs (fruits, fruit juice, dairy products, chocolate, honey, mushrooms and chewing gum) that affect the gastrointestinal permeability evaluation before the main trials and they were asked to avoid consuming these food items on day 5 in the present study. On the morning of day 6, participants reported to the laboratory at 0845 after a 12-h overnight fast (no food or drink after 2100 on day 5) in both trials. No drinking water was permitted after 0645 on the morning of day 6 in either trial. Drinking water and a standardized meal was provided to each participant during a 6-h laboratory-based experiment on day 6 and this was standardized between trials as it affects gastrointestinal permeability evaluations (further details are provided under “Evaluation of gastrointestinal permeability”). Body mass was measured, and a rectal probe was inserted to measure the rectal temperature during exercise using a thermistor (401 J, Nikkiso-Therm Co., Ltd., Tokyo, Japan). After 15 min, a fasting venous blood sample was collected in a seated position by venepuncture at 0900. Further venous blood samples were collected immediately post-exercise (1045), and 30 min (1115), 1 h (1145), 2 h (1245) and 4 h (1445) post-exercise. 15 min after collecting fasting blood samples (at 0915), participants remained seated in a chair and ingested their designated supplements [i.e., placebo (2.46 g of maltodextrin) or cystine and glutamine (0.23 g of L-cystine, 1.00 g of l-glutamine, and 1.23 g of maltodextrin)] with 200 mL of warm water (37 °C). After 30 min of rest, participants performed running exercises at a speed corresponding to 75% of maximal oxygen uptake for 1 h. The atmospheric temperature and relative humidity during the 1-h exercise session was maintained at 25 °C and 60%, respectively, in both trials. Heart rate was monitored throughout exercise using short-range telemetry (Polar RCX3, Polar Electro, Kempele, Finland), and ratings of perceived exertion were periodically assessed [16]. Thereafter, participants were asked to remain seated (reading, writing, or working on a computer) in the laboratory until 1445. The mean temperature and humidity during the experimental trials were 25.4 ± 1.1 °C and 56.3 ± 3.9% (mean ± SD), respectively.

### Evaluation of gastrointestinal permeability

Gastrointestinal permeability was evaluated by analysing the plasma samples using the liquid chromatography-tandem mass spectrometry method based on previously published methods [19]. Participants consumed a standardized, dual-sugar solution containing 10 g of lactulose and 5 g of mannitol in 100 mL of warm water (37 °C) and 200 mL of warm water (37 °C) 20 min after the start of running in each exercise trial in the main experiment (1005) or rest (i.e., the baseline gastrointestinal permeability evaluation) in the preliminary test. Participants were asked to ingest 200 mL of warm water (37 °C) immediately post-exercise (1045) and 1 h post-exercise (1145). Thereafter, participants were asked to ingest 500 mL of room temperature water (25 °C), and this volume was asked to be consumed until the end of each main trial (1445). The timing and volumes of ingestion mimicked the baseline (i.e., resting) gastrointestinal permeability evaluation conducted in the preliminary test. The plasma samples were deproteinized with acetonitrile after adding stable isotope labels as internal standard substances. The prepared samples were quantitated using ultra-high performance liquid chromatography with electrospray ionization tandem mass spectrometry (Nexera, Shimadzu Co., Kyoto, Japan; QTRAP 5500, AB Sciex Pte. Ltd., Tokyo, Japan). The analysis method was validated concerning the bioanalytical method validation guidelines [20]. The intra-assay coefficients of variation were 9.3% for lactulose and 10.4% for mannitol. The validity of the measured values was confirmed by the quality control sample, and the criteria were 100 ± 15%.

### Evaluation of gastrointestinal damage

Plasma I-FABP concentrations were determined using EDTA plasma via enzyme-linked immunosorbent assay kits (HK406, Hycult Biotech Inc., Pennsylvania, USA). All analyses for each participant were completed within the same run for each measure. The intra-assay coefficient of variation was 4.4%. We planned to analyze absolute values. However, since there was a large inter-participant variability in plasma I-FABP in the present study as was the case in a previous study [21], we presented plasma I-FABP concentration as changes compared with fasting (0900) values.

### Other blood analyses

For fasting serum triglycerides (TG), non-esterified fatty acids (NEFA), total cholesterol (TC), low-density lipoprotein cholesterol (LDL-C) and high-density lipoprotein cholesterol (HDL-C) measurements, venous blood samples were collected into tubes containing clotting activators to isolate serum. Thereafter, samples were allowed to clot for 30 min at room temperature (i.e., 25 °C) and were centrifuged at 1861×*g* for 10 min at 4 °C. Serum was removed, aliquoted and stored at − 80 °C for later analysis. For fasting plasma glucose measurements, venous blood samples were collected in tubes containing sodium fluoride-EDTA. Thereafter, both tubes were immediately centrifuged and treated as per the aforementioned procedure. Enzymatic colorimetric assays were used to measure serum TG (Pureauto S TG-N, Sekisui Medical Co., Ltd., Tokyo, Japan), NEFA (NEFA-HR, Wako Pure Chemical Industries, Ltd., Osaka, Japan), TC (T-cho Determiner L TC II, Hitachi Chemical Diagnostics Systems Co., Ltd., Tokyo, Japan), LDL-C (Matabo Lead LDL-C, Hitachi Chemical Diagnostics Systems Co., Ltd.), and HDL-C (Matabo Lead HDL-C, Hitachi Chemical Diagnostics Systems Co., Ltd.) and plasma glucose (GLU-HK(M), Shino-Test Corporation, Tokyo, Japan). Analyses for each participant were completed within the same run for each measure. Intra-assay coefficients of variation were 0.3% for TG, 0.8% for NEFA, 0.6% for TC, 0.8% for LDL-C, 1.2% for HDL-C and 0.4% for glucose.

### Sample size calculations and statistical analyses

The sample size was set to 14 based on our pilot study and a previous study [6]. The validity of this sample size was examined by calculating the statistical power. The sample size was validated using L:M as the primary study outcome, using the same sample size calculation methods used in a previous crossover study [6]. Analyses were performed using G*Power 3.1.0 [22]. Based on a pilot study with ten participants, we calculated that an estimated total sample size of 14 was needed to provide 88% power to detect between-trial differences for two trials, with an alpha level set at 0.05 and a correlation of 0.5. This sample size estimation was powered to detect an effect size of 0.91 (Cohen’s d), using a paired t-test for comparison between trials. We also calculated the required sample size based on previous data [6]. The study reported within-subject effects (trial: glutamine vs. placebo; effect size, Cohen’s *d* = 0.83 for the plasma lactulose to rhamnose ratio) using glutamine versus the placebo response to a 60-min treadmill run [6]. The sample size was calculated to detect an effect size of 0.83 using a paired *t*-test for comparison between trials. For two trials with an alpha level set at 0.05 and a correlation of 0.5, an estimated total sample size of 14 would provide 82% power to detect between-trial differences. Based on these calculations, we recruited 16 participants to allow for potential withdrawals. Data were analyzed using *R* (version 3.4.3; R Foundation for Statistical Computing, Vienna, Austria), while linear mixed models were analyzed using the lme4 and lmerTest libraries in R. The full analysis set was based on the intention-to-treat concept. A linear mixed model for repeated measures [trial (placebo or amino acids), time (treated as categorical with levels at 0, 30, 60, 120 and 240 min post-exercise], factors related to a crossover study design (i.e., term and sequence) as fixed factors, and participants as the random factor were used for each dependent variable. Statistical significance was accepted at a two-sided 5% level. Results are reported as mean ± SD. However, in the linear mixed models for longitudinal data, the results for the trial are reported as coefficient ± standard error (SE), with the notation “coefficient ± SE” to distinguish them from the mean ± SD.

## Results

### Fasting biochemistry parameters

Serum TG, NEFA, TC, LDL-C and HDL-C and plasma glucose concentrations measured on the morning of day 6 for each trial, are shown in Table 1. The fasting concentrations of all parameters did not differ between the trials.

**Table 1:** Fasting blood parameters measured on the morning of day 6 in the cystine and glutamine supplementation (Cys2/Gln) and placebo trials

	Cys2/Gln	Placebo	*P* value
TG (mmol/L)	1.09 ± 0.37	1.08 ± 0.35	0.954
NEFA (mmol/L)	0.38 ± 0.24	0.45 ± 0.33	0.262
TC (mmol/L)	4.89 ± 0.55	4.82 ± 0.60	0.285
LDL-C (mmol/L)	2.83 ± 0.45	2.66 ± 0.63	0.098
HDL-C (mmol/L)	1.46 ± 0.31	1.53 ± 0.24	0.160
Glucose (mmol/L)	4.92 ± 0.39	4.77 ± 0.40	0.144

Values are means ± SD for *n *= 16. Values were compared using paired *t*-tests

*TG* triglycerides, *NEFA* non-esterified fatty acids, *TC* total cholesterol, *LDL-C* low-density lipoprotein cholesterol, *HDL-C* high-density lipoprotein cholesterol. *P* values were calculated by linear mixed effect models adjusted for crossover study design factors (i.e., term and sequence)

### Responses during running

No significant differences were observed between the placebo, and cystine and glutamine supplementation trials regarding mean heart rate (placebo: 157 ± 12 beats/min; cystine and glutamine: 158 ± 13 beats/min, linear mixed model; *P* = 0.151), relative exercise intensity (placebo: 78.3 ± 5.8% of maximum oxygen uptake; cystine and glutamine: 78.9 ± 5.7% of maximum oxygen uptake, linear mixed model; *P* = 0.168), or peak rectal temperature (placebo 38.3 ± 0.8 °C; cystine and glutamine: 38.4 ± 0.5 °C, linear mixed model; *P* = 0.686).

### Plasma L:M

**Fig. 2:**
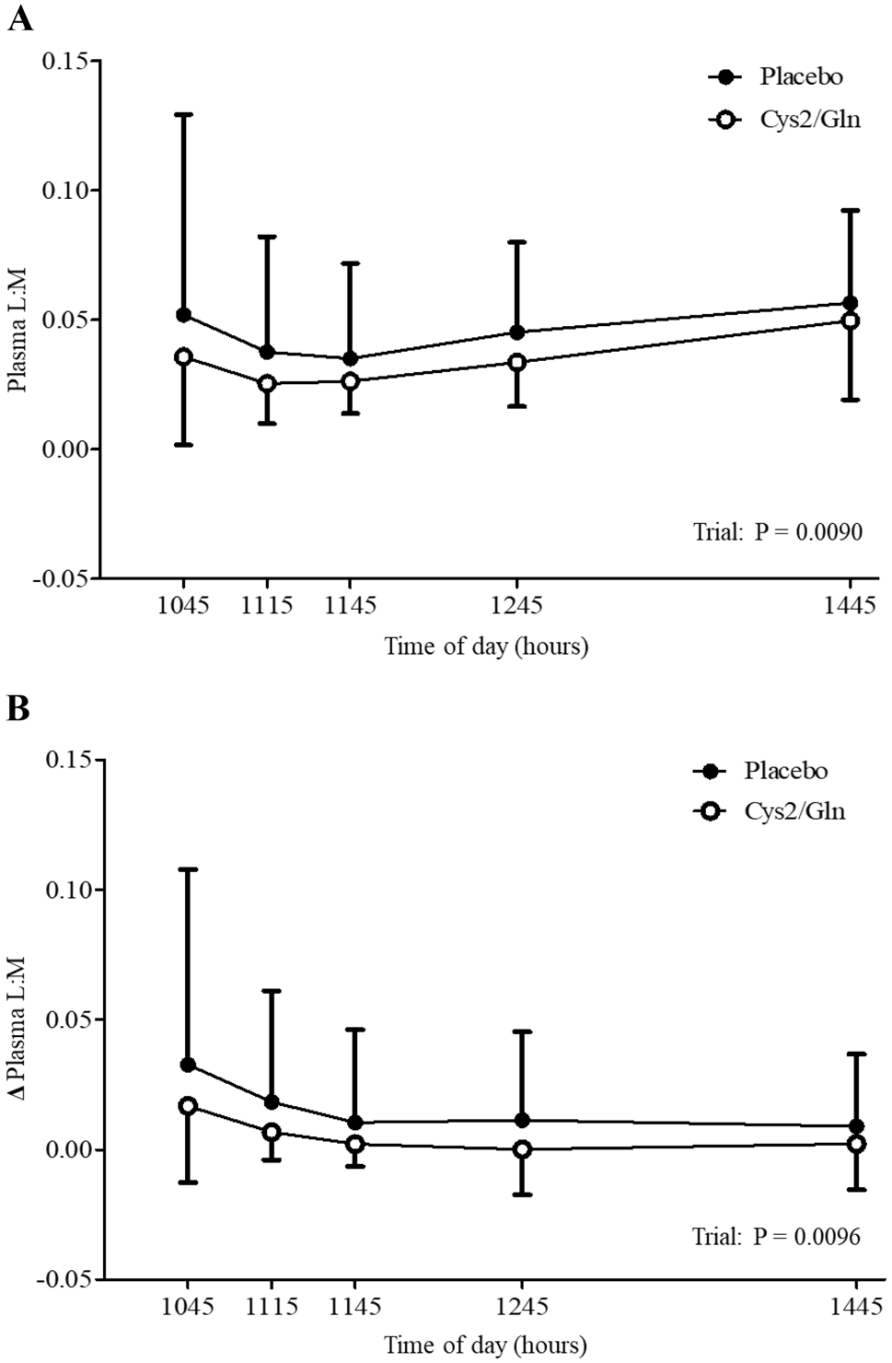
Absolute (**A**) and delta (**B**) plasma lactulose to mannitol ratio (L:M) in the cystine and glutamine supplementation (Cys2/Gln) and placebo trials. Data are means ± SD for *n* = 16. Data were analyzed using a linear mixed model

The absolute plasma L:M ratio is shown in Fig. 2A. Plasma L:M was lower in the cystine and glutamine supplementation trial than in the placebo trial (linear mixed model, coefficient ± SE; − 0.011 ± 0.004, *P* = 0.0090). However, increased plasma L:M in the cystine and glutamine supplementation trial was observed at 120 min and 240 min after exercise. Given that this trend was also observed in the resting state (i.e., the baseline gastrointestinal permeability evaluation), an additional analysis was performed using the delta values (i.e., the change from the value in the resting state). In this additional analysis, plasma L:M was lower in the cystine and glutamine supplementation trials compared to that in the placebo trial (linear mixed model, coefficient ± SE; -0.011 ± 0.004, P = 0.0096) (Fig. 2B).

### Plasma I-FABP

**Fig. 3:**
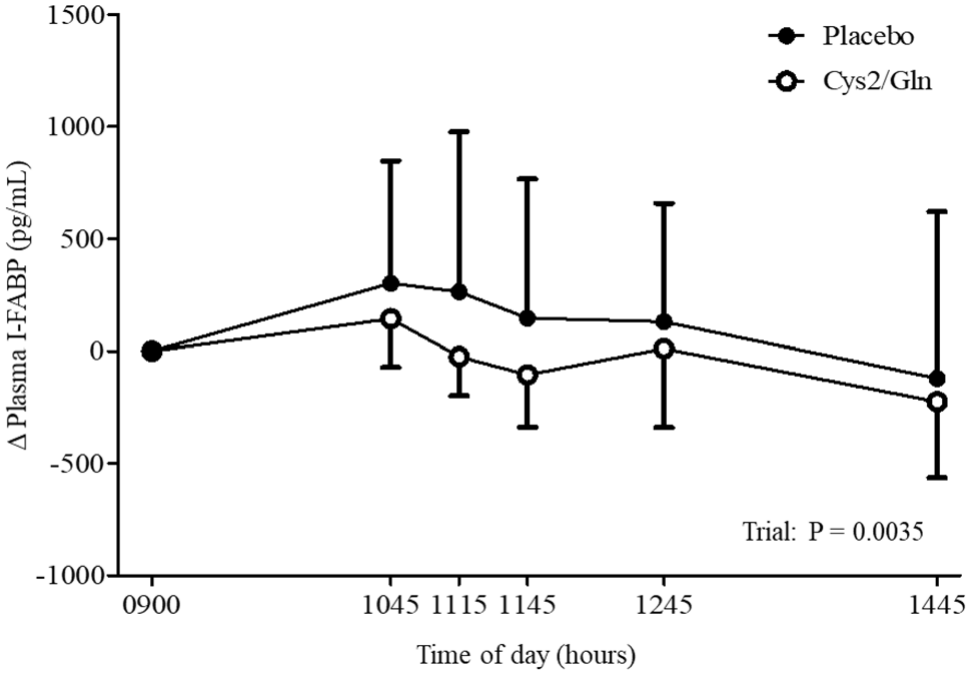
Delta plasma intestinal fatty acid-binding protein (I-FABP) in the cystine and glutamine supplementation (Cys2/Gln) and placebo trials. Data are means ± SD for *n* = 16. Data were analyzed using a linear mixed model

Delta [by subtracting the value at each time point from the baseline value (i.e., 0900)] plasma I-FABP concentrations are shown in Fig. 3. Plasma I-FABP concentrations were lower in the cystine and glutamine supplementation trial than in the placebo trial (linear mixed model, coefficient ± SE [pg/mL]; − 195.3 ± 65.7, *P* = 0.0035).

## Discussion

The present study demonstrated that compared with ingestion of maltodextrin, oral cystine and glutamine supplementation attenuated the exercise-induced increase in plasma L:M and I-FABP concentrations in young active men. A small amount of cystine (0.69 g/day from days 1–5, and 0.23 g/day on day 6) and glutamine (3.00 g/day from days 1–5, and 1.00 g/day on day 6) were used in the present study, compared with the amounts of amino acids used in previous studies [i.e., ranging from 0.25 g/kg fat-free mass (FFM) to 0.9 g/kg FFM (or /day)] [6, 7, 9, 23]. Our findings may, therefore, underscore the practical importance of nutritional strategies to reduce gastrointestinal problems in real-life settings where athletes, endurance athletes in particular, often experience such complications, both during and after prolonged strenuous exercise [24].

Previous studies investigated the potential role of nutritional supplement interventions for the prevention or attenuation of exercise-induced gastrointestinal permeability markers in response to exercise under conditions of exercise-heat stress or field competitive events in humans—these were conducted with various supplements, including antioxidants, amino acids (i.e., L-arginine and glutamine), bovine colostrum, curcumin, and probiotics (for a review of these, see Reference [5]). Three of these studies reported reductions in the markers of gastrointestinal permeability [6, 9, 25]. Of these three studies, two studies used glutamine and were characterized by a similar duration (i.e., for 7 days or immediately pre-exercise) and/or amount of supplements (0.9 g/kg FFM/day or 0.25, 0.5 and 0.9 g/kg FFM) used in the present study [6, 9]. Furthermore, regarding the dosage of glutamine and the magnitude of response in gastrointestinal permeability markers after exercise, one study has reported that 0.9 g/kg FFM/day of glutamine administered for 7 days attenuated the increases in gastrointestinal permeability by approximately 55% compared to placebo [6]. Another study, in which oral glutamine was consumed 2 h before running, has also reported reduced gastrointestinal permeability by approximately 25% with 0.25 g/kg FFM of glutamine and by approximately 40% with 0.9 g/kg FFM of glutamine at 60 min post-exercise compared to placebo [9]. In the present study, increased gastrointestinal permeability (i.e., a mean of 4 h after exercise) was found to be attenuated by approximately 66% with oral cystine and glutamine supplementation compared to the placebo trial. Also, it is worth noting that these two studies [6, 9] conducted exercises at a relatively higher temperature (30 °C) than the present study (25 °C). Nonetheless, given that there is a positive relationship between increased core body temperature (induced by intense and prolonged exercise in a hyperthermic environment) and increased gastrointestinal permeability [1, 3], the present study also observed an increase in core body temperature to 38 °C (i.e., on average in both trials). This effectively attenuated the increases in gastrointestinal permeability and damage by oral cystine and glutamine supplementation. Indeed, our findings are consistent with those of previous studies [21, 26–28] where the environmental conditions and/or exercise volumes were similar to those of the present study although various supplements (i.e., bovine colostrum, zinc and probiotics) were used in these previous studies. However, inconsistent results have also been reported regarding its effectiveness in lowering gastrointestinal permeability and intestinal cellular damage [29–31]. The duration of exercise, dosage of the saccharide used in a permeability test, nutritional status before the experiment (i.e., a fasted or fed state), timing of a saccharide drink ingestion (i.e., before or after exercise) and environmental conditions are possible reasons for the inconsistent findings among studies as these are confounding factors affecting the gastrointestinal permeability and damage [3]. Additional research with a standardized protocol is required to elucidate the impact of supplementation on gastrointestinal permeability and damage in response to exercise in various environments.

The mechanisms underlying the exercise-induced increase in gastrointestinal permeability and intestinal cellular damage are unclear, but are likely mediated by an increase in core temperature during or shortly after strenuous exercise, and/or a reduced total splanchnic perfusion, causing gastrointestinal ischemia [3, 4]. Glutamine intake in response to running (i.e., 60 min at 65–70% of maximal oxygen uptake) has been shown to stimulate heat shock protein 70 and nuclear factor of kappa light polypeptide gene enhancer in B-cells inhibitor alpha in human peripheral blood mononuclear cells, which leads to inactivation of the nuclear translocation of nuclear factor-κB proinflammatory pathway [6]. A recent in vitro study also suggested that cystine reduces barrier dysfunction caused by oxidative stress and improves intestinal barrier function [13]. It is not possible to determine which of these mechanisms operated in the present study, but mixed supplementation of cystine and glutamine may have contributed to facilitating these mechanisms and attenuating exercise-induced increases in plasma L:M and I-FABP concentrations. Despite the effectiveness of supplementation for the prevention of increased gastrointestinal permeability and damage in response to exercise, though not all [23], glutamine supplementation did not appear to influence subjective symptoms of gastrointestinal discomfort in response to heat [9]. Therefore, further studies are needed to determine the effect of supplementation, if any, on the subjective symptoms of gastrointestinal discomfort in a real-world setting (i.e., repeated exercise training).

The strength of the present study includes the protective effect of intestinal permeability and damage were observed at relatively low mixed amino acid doses. Indeed, most previous studies using glutamine as a nutritional intervention have employed relatively large doses of glutamine (range from 0.25 g/kg FFM to 0.9 g/kg FFM/day) [5]. Thus, our supplementation dose is more accessible to consume on a daily basis. There are limitations to the present study that should be addressed. Our findings were not able to provide potential mechanisms for the attenuation of the exercise-induced increase in plasma L:M and I-FABP concentrations since the present study was not focused on or designed with a mechanistic intent. In addition, the present study was designed to explore the effect of prior amino acid supplementation on markers of gastrointestinal permeability and damage in response to an acute bout of prolonged strenuous exercise in active men. Thus, the effectiveness of post-exercise supplementation on exercise-induced gastrointestinal syndrome in trained athletes is not known. Future research is required for a longer observational period than the protocol used in the present study to observe the favourable effects, if any, on post-exercise nutritional strategies in the presence of increased gastrointestinal permeability and damage or gastrointestinal syndrome in this population [3].

## Conclusion

In conclusion, oral cystine and glutamine supplementation attenuated L:M and plasma I-FABP concentrations in response to 1 h of strenuous running in young active men, suggesting a transient reduction in gastrointestinal permeability and damage via amino acid supplementation. These findings require further investigation over a longer period with a more practical setting in athletes who experience gastrointestinal problems through habitual exercise training.

## Data Availability

Some or all data sets generated during and/or analyzed during the present study are not publicly available but are available from the corresponding author on reasonable request.

## Abbreviations

Cys2/Gln: Cystine and glutamine supplementation
HDL-C: High-density lipoprotein cholesterol
I-FABP: Intestinal fatty acid-binding protein
LDL-C: Low-density lipoprotein cholesterol
L:M: Lactulose to mannitol
NEFA: Non-esterified fatty acids
SD: Standard deviation
SE: Standard error
TC: Total cholesterol
TG: Triglycerides
